# Refractory ascites and graft dysfunction in early renal transplantation

**DOI:** 10.1590/2175-8239-JBN-2018-0175

**Published:** 2019-03-18

**Authors:** Catarina Pereira Eusébio, Sofia Correia, Filipa Silva, Manuela Almeida, Sofia Pedroso, La Salete Martins, Leonídio Diais, José Queirós, Helena Pessegueiro, Ramon Vizcaíno, António Castro Henriques

**Affiliations:** 1 Serviço de Nefrologia do Centro Hospitalar de Trás-os-Montes e Alto Douro, Vila Real, Portugal.; 2 Serviço de Nefrologia e Transplantação Renal do Centro Hospitalar do Porto, Porto, Portugal.; 3 Serviço de Gastroenterologia do Centro Hospitalar do Porto, Porto, Portugal.; 4 Serviço de Anatomia Patológica do Centro Hospitalar do Porto, Porto, Portugal.

**Keywords:** Kidney Transplantation, Ascites, Fibrosis, Pharmaceutical Preparations., Transplante de Rim, Ascite, Fibrose, Preparações Farmacêuticas.

## Abstract

The occurrence of ascites after Renal Transplant (RT) is infrequent, and may be a consequence of surgical or medical complications. Case report: 61 year-old, male, history of arterial hypertension, tongue carcinoma and alcoholic habits 12-20g/day. He had chronic kidney disease secondary to autosomal dominant polycystic kidney disease, without hepatic polycystic disease. He underwent cadaver donor RT in September 2017. He had delayed graft function by surgically corrected renal artery stenosis. He was admitted in January 2018 for ascites de novo, with no response to diuretics. HE had visible abdominal collateral circulation. Graft dysfunction, adequate tacrolinemia, Innocent urinary sediment, mild anemia, without thrombocytopenia. Serum albumin 4.0g / dL. Normal hepatic biochemistry. Peritoneal fluid with transudate characteristics and serum albumin gradient > 1.1. Ultrasound showed hepatomegaly, permeable vascular axes, without splenomegaly. Mycophenolate mofetil was suspended, with reduced remaining immunosuppression. He maintained refractory ascites: excluded infectious, metabolic, autoimmune and neoplastic etiologies. No nephrotic proteinuria and no heart failure. MRI: micronodules compatible with bile cysts. Upper Digestive Tract Endoscopy did not show gastroesophageal varicose veins. Normal abdominal lymphoscintigraphy. He underwent exploratory laparoscopy with liver biopsy: incomplete septal cirrhosis of probable vascular etiology some dilated bile ducts. He maintained progressive RT dysfunction and restarted hemodialysis. The proposed direct measurement of portal pressure was delayed by ascites resolution. There was further recovery of the graft function. Discussion: Incomplete septal cirrhosis is an uncommon cause of non-cirrhotic portal hypertension. Its definition is not well known, morphological and pathophysiological. We have not found published cases of post-RT ascites secondary to this pathology, described as possibly associated with drugs, immune alterations, infections, hypercoagulability and genetic predisposition.

## Resumo

A ocorrência de ascite no pós-Transplante Renal (TR) é infrequente, podendo ser consequência de complicações cirúrgicas ou médicas. Caso clínico: 61 anos, masculino, antecedentes de hipertensão arterial, carcinoma da língua e hábitos alcoólicos 12-20g/dia. Doença renal crônica secundária à doença renal poliquística autossômica dominante, sem poliquistose hepática. Submetido a TR de doador cadáver em setembro de 2017. Atraso na função de enxerto por estenose da artéria renal, corrigida cirurgicamente. Internado em janeiro de 2018 por ascite de novo, sem resposta a diuréticos. Circulação colateral abdominal visível. Disfunção do enxerto, tacrolinemia adequada. Sedimento urinário inocente. Anemia ligeira, sem trombocitopenia. Albumina sérica 4,0g/dL. Bioquímica hepática normal. Líquido peritoneal com características de transudado e gradiente sero-ascítico de albumina > 1,1. Ecografia com hepatomegalia, eixos vasculares permeáveis, sem esplenomegalia. Suspendeu micofenolato mofetil, reduziu restante imunossupressão. Manteve ascite refratária: excluídas etiologias infecciosas, metabólicas, autoimunes e neoplásicas. Sem proteinúria nefrótica e sem insuficiência cardíaca. RM: micronódulos compatíveis com quistos biliares. EDA sem varizes gastroesofágicas. Linfocintigrafia abdominal normal. Submetido a laparoscopia exploradora com biópsia hepática: cirrose septal incompleta de provável etiologia vascular, alguns ductos biliares dilatados. Manteve disfunção progressiva do TR, reiniciou hemodiálise. Proposta medição direta da pressão portal, protelada por resolução da ascite. Recuperação posterior da função de enxerto. Discussão: A cirrose septal incompleta é uma causa incomum de hipertensão portal não cirrótica. A sua definição é morfológica e a fisiopatologia, pouco conhecida. Não encontramos publicados casos de ascite pós-TR secundária a esta patologia, descrita como possivelmente associada a fármacos, alterações imunitárias, infecções, hipercoagulabilidade e predisposição genética.

## Introduction

The term "ascites" refers to a pathological buildup of fluid in the peritoneal cavity. It is associated with symptoms and changes in the physical examination when the volume is greater than 1.5 L, with smaller volumes being diagnosed through image studies.^1^^,^ 2 In its etiology, conditions that directly involve the peritoneum (infection, neoplasia) may be a consequence of changes in another organ, or systemic changes. In the West, cirrhosis is the main cause of ascites (75%), followed by peritoneal neoplasia (12%), heart failure (5%) or peritoneal tuberculosis (2%).^3^ Ascites can also be classified as associated with portal hypertension (such as liver cirrhosis, heart failure or Budd-Chiari syndrome) or not associated with portal hypertension (peritonitis or peritoneal metastases).^4^ Another etiology, uncommon in Western countries, is idiopathic non-cirrhotic portal hypertension, and it is rarely considered in the differential diagnoses.^5^ Little is known about the pathophysiology of this entity, which is defined by the presence of clinical signs and symptoms of portal hypertension in the absence of known cirrhosis or liver disease.^6^

In a patient with ascites as its initial clinical condition, one should perform a detailed clinical history and physical examination as well as analytical and image assesments.^2^ Diagnostic paracentesis is the test that alone offers more information and should be performed early.^1^ A serum albumin (SA) greater than 1.1 g/dL has 97% diagnostic accuracy for ascites secondary to portal hypertension.^2^^,^ 3

The occurrence of ascites in the post-renal transplantation is rare and may occur with either preserved graft function or in situations of its dysfunction.^4^ There are reports of cases associated with problems such as rejection, graft decapsulation, urinary or vascular leakage, lymphocytosis, transudation, or infection.^7^^,^ 8 In rare cases, ascites and hepatotoxicity is associated with immunosuppressive drugs such as mycophenolate mofetil, azathioprine and sirolimus.^7^^,^ 9 ^,^ 10 Nephrogenic ascites should also be considered in cases of advanced or terminal renal dysfunction. Its pathophysiology is unknown, but it is assumed that there is an increase in the permeability of the peritoneal membrane with consequent exudation, characterized by SA less than 1.1 g/dL.^11^

## Clinical case

A 61-year-old male, with a history of arterial hypertension, tongue carcinoma, had been submitted to partial glossectomy 8 years earlier. He had inactive smoking habits and alcoholic habits of about 12-20 g/day. Personal and family history of chronic renal disease secondary to dominant Autosomal Polycystic Kidney Disease, with no hepatic impairment diagnosed. He was in hemodialysis for four years, without adversities. He underwent nephrectomy of the right kidney in preparation for renal transplantation (RT). No known history of blood transfusion or hypersensitizing events. He underwent RT from a cadaver donor four months before the current situation, with three HLA Class I and one Class II incompatibilities, without anti-doping antibodies. Donor and recipient with previously acquired immunity to CMV (IgG positive for both serologies).

He underwent induction immunosuppression with Basiliximab, tacrolimus, mycophenolate mofetil (MMF) and methylprednisolone. His delayed graft function by renal artery stenosis was surgically corrected in the immediate post-RT. At the time, he had high serum creatinine (CrS) 2.6 mg/dL. No need for transfusion of erythrocyte concentrate in the post-RT period. She maintained outpatient follow-up, with basal CrS of 2.6-2.8 mg/dL. He had been regularly medicated with tacrolimus, prednisolone, mycophenolate mofetil, pantoprazole, furosemide, amlodipine, carvedilol, lisinopril, tamsulosin, folic acid, B vitamins and erythropoietin analogue. He was hospitalized as a result of ascites again, progressive aggravation, without response to an increase in diuretic therapy. He had no dyspnea, no orthopnea, had visible abdominal collateral circulation and a slight peripheral edema on physical examination, with no other noticeable changes. Analytically, the graft function worsened (CrS 4.5 mg/dL), with adequate tacrolimus levels (Tacrolimus 8.9 ng/mL). Light anemia (Hb 10.7 g/dL), no thrombocytopenia (platelets 154,000). Serum albumin 4.0 g/dL, LDH 266 U/L. Unchanged hepatic biochemistry (TGO 24 U/L, TGP 15 U/L, GGT 32 U/L, alkaline phosphatase 55 U/L, total bilirubin 0.25 mg/dL); normal coagulation panel. Innocent urinary sediment, with a P/CrU ratio of 0.33. The patient was submitted to diagnostic and evacuation paracentesis, with peritoneal fluid drainage (PF), with a slightly milky aspect but no diagnostic cellularity of peritonitis, with biochemical transudate characteristics (total protein and LDH), SA > 1.1 g/dL and triglycerides in the upper limit of normality (210 mg/dL, the upper limit being 200 mg/dL), which could justify the macroscopic appearance of PF. Abdominal ultrasound showed hepatomegaly (16.3 cm; patient height 165 cm), with liver with regular contours and increased parenchyma echogenicity, pervious vascular axes and no splenomegaly. He suspended MMF and reduced the remaining immunosuppression. He maintained refractory ascites, requiring frequent evacuation paracentesis and drainage higher than 3 L (administered i.v. albumin when ≥ 5 L).

Graft dysfunction was interpreted in the context of effective intravascular volume depletion by losses to the third space and marked increase of intra-abdominal pressure (high volume ascites under tension), which is corroborated by the transient improvement in graft function, objectified immediately after performing evacuation paracenteses (CrS minimum 3.1 mg/dL). The results of the PF microbiological, mycological and mycobacteriological exams were negative. Viral hepatitis and HIV serologies were negative. He had negative alpha-fetoprotein, copper, ceruloplasmin, alpha-1-antitrypsin and hepatic autoimmunity markers. His ferritin was slightly increased (467 mg/dL). He also had peripheral blood flow cytometry and normal PF. He showed negative stool parasitological examination. His contrast CT scan revealed scattered millimetric hypervascular hepatic nodules and small simple biliary cysts. His MRI with hepato-specific contrast showed multiple micronodules with features suggestive of biliary cysts. His upper gastrointestinal endoscopy showed no gastroesophageal varices. His abdominal lymphoscintigraphy had no changes, and his transthoracic echocardiogram showed no relevant changes.

**Figure 1:**
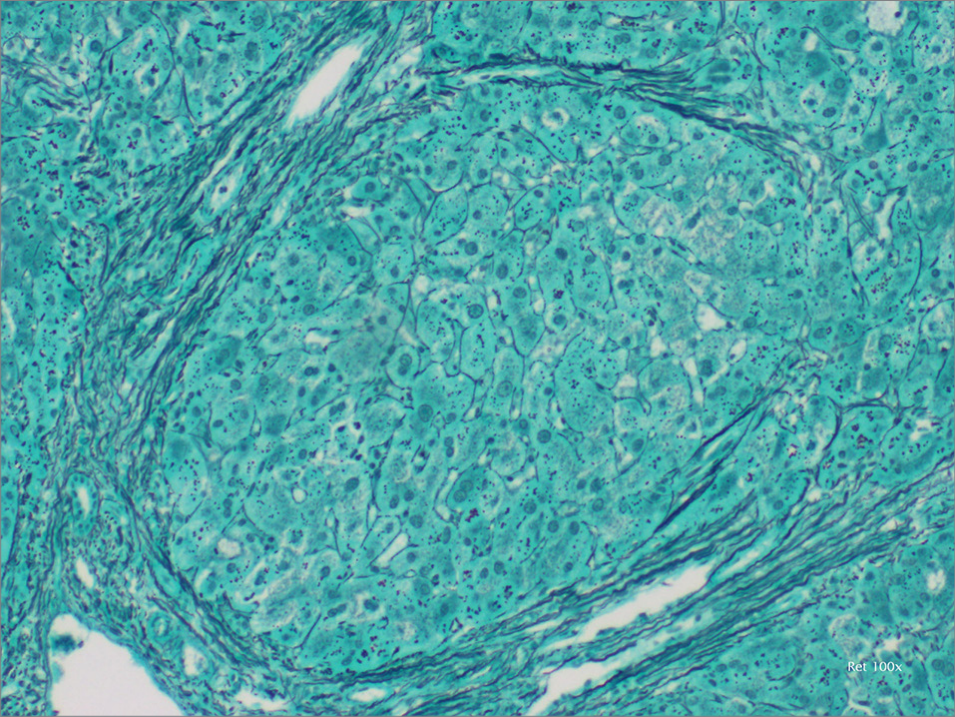
Liver histology. Reticulin staining 100x. Fibrous septa are observed which incompletely resemble a nodule.

**Figure 2:**
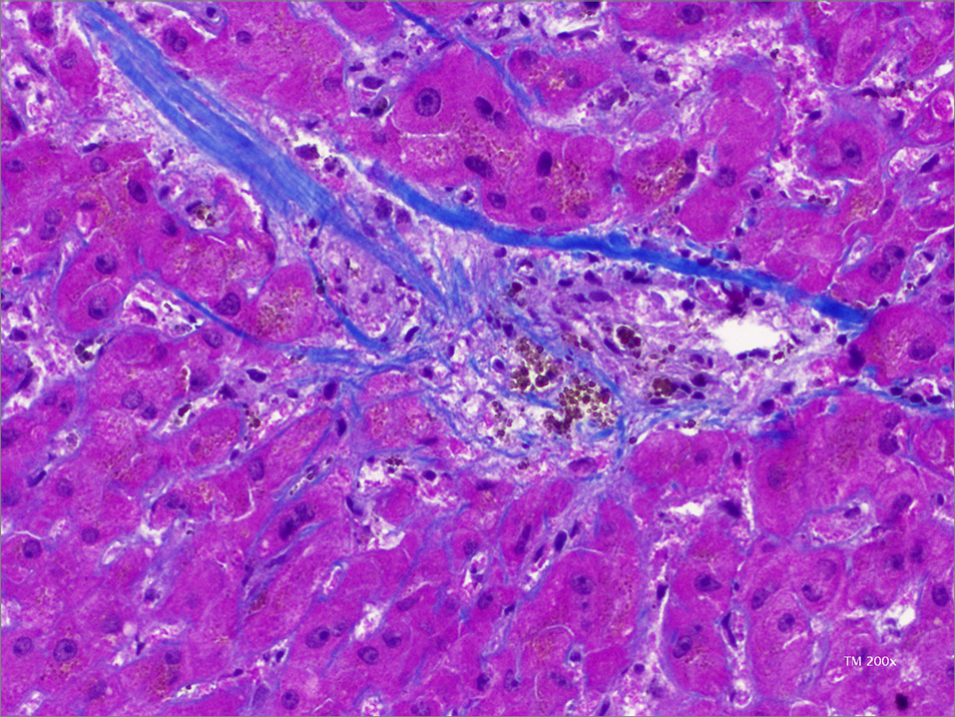
Liver histology. Masson trichrome 200x. We see central lobular vein with changes suggestive of veno-occlusive disease.

We discussed this case at a multidisciplinary team meeting: due to the lack of diagnostic data, exploratory laparoscopy was suggested. Intraoperatively, the liver had a nodular macroscopic appearance. In the same surgical procedure, we performed a surgical wedge biopsy. The anatomopathological exam revealed incomplete septal cirrhosis of probable vascular etiology (veno-occlusive disease) and dilated bile ducts in some portal spaces (Figures 1 and 2). The patient evolved with progressive RT dysfunction and required regular hemodialysis. In a later gastroenterology consultation, a direct measurement of portal pressure was proposed, with a view to the eventual placement of a transjugular intrahepatic portal-systemic shunt, which was, however, delayed because of the complete ascites resolution. Given the favorable evolution, the hypercoagulability disorder study was also not performed. After one month on regular dialysis, the patient recovered graft function and maintained stable RT function, with CrS 1.9mg/dL, under double immunosuppression (tacrolimus and prednisolone). At 6 months of follow-up, the ascites did not recur.

## Discussion

The term incomplete septal cirrhosis (ISC) was first introduced by Popper in 1966.^12^ Liver biopsy studies revealed a ISC frequency of 0.74-1.4%.^13^ Histologically, it is characterized by of hepatic parenchyma nodularity, vascularized septa, reticulin buildup between hyperplastic parenchyma zones, sinusoidal dilatation and hepatocyte hyperplasia.^14^ One of the pathophysiological mechanisms suggested was the existence of obliterative portal venopathy, with consequent heterogeneity in the hepatic parenchyma portal irrigation.

ISC is one of the histological presentation patterns of idiopathic non-cirrhotic portal hypertension (INCPH), and it is considered by several authors as a stage of disease manifestation.^12^^,^ 15 The international nomenclature of INCPH is ambiguous. In Asia, where it is most frequent, it is known as non-cirrhotic portal fibrosis (India) and idiopathic portal hypertension (Japan). In the West, it has been dubbed hepatorenal sclerosis, idiopathic portal hypertension, incomplete septal cirrhosis, and nodular regenerative hyperplasia.^15^

The main etiologies proposed for INCPH are: infectious (bacterial infections of the gastrointestinal tract and umbilical pyemia with repeated septic embolization for portal circulation, schistosomiasis and HIV); (systemic sclerosis, systemic lupus erythematosus, hypogammaglobulinemia); exposure to drugs and toxins (arsenic, azathioprine); prothrombotic states.^16^ Still, there are rare forms of genetic and familial predisposition described.^14^^,^ 15

The diagnostic criteria for INCPH are the presence of a clinical sign of portal hypertension (splenomegaly/hyperpesplenism, esophageal varicose veins, ascites, increase in the hepatic venous pressure gradient or presence of portal venous collaterals), after cirrhosis, vascular thrombosis of the portal and hepatic veins, and conditions that may be associated with chronic liver disease (infectious, autoimmune, metabolic, etc.).^15^^,^ 17

In the clinical case described here, the appearance of ascites seems to be temporally related to RT. Therefore, late surgical complications were excluded by imaging exams. The PF biochemical analysis revealed a SA > 1.1 g/dL, which is suggestive of portal hypertension and is corroborated by the presence of exuberant collateral venous circulation in the abdominal wall, and hepatomegaly. Although splenomegaly and esophageal varices did not coexist, the clinical picture, at the time of diagnosis, showed little evolution time, which may justify the absence of these findings. A SA > 1.1 g/dL makes unlikely etiologies such as ascites secondary to infectious, nephrogenic or neoplastic peritonitis, which were also investigated and ruled out. The intra-abdominal vascular axes thrombosis, hepatic cirrhosis or liver disease (metabolic, autoimmune and viral), as well as heart failure or nephrotic syndrome that could justify the clinical picture.

The authors thus reached the histological diagnosis of incomplete septal cirrhosis after exploratory laparoscopy and surgical liver biopsy. The main causes that are thought to be associated with this entity were described above and also mostly excluded during the diagnostic investigation. However, it remains the hypothesis that the ISC, in the case of our patient, may be associated with the pharmacological exposure to the MMF. There is only one reported case of refractory ascites in the literature, after kidney-pancreas transplantation, which association with mycophenolic acid was confirmed after ascites resurgence of with its reintroduction. However, in this case, no liver biopsy was performed.^9^

Veno-occlusive disease, which has been described as a possible etiology of hepatic septal cirrhosis. In our clinical case, it is characterized by loss of sinusoidal wall integrity and obliterative venulitis, and has been widely described as associated with myeloablative regimens used in hematopoietic cell transplantation. However, it has also been described as secondary to other chemotherapy, radiotherapy, teas/herbal products and other pharmacological therapies, among which the antimetabolite drug Azathioprine is highlighted, with interest in our case.^18^^,^ 19

In our patient, after one month in a regular hemodialysis program, we achieved ascites resolution and graft function recovery. After 6 months of follow-up, with double immunosuppression (prednisolone and tacrolimus [low tacrolinemia, 5-6 ng/mL]), the patient had no evidence of ascites relapse, confirmed by imaging, which is more in favor of the possible association between MMF, refractory ascites and incomplete septal cirrhosis/ INCPH, described in this clinical case.

## References

[1] Sood R (2004). Ascites: Diagnosis and Management. J Indian Acad Clin Med.

[2] Tsochatzis EA, Gerbes AL (2017). Diagnosis and treatment of ascites. J Hepatol.

[3] Garcia-Tsao G, Dooley JS, Lok ASF, Burroughs AK, Heathcote MB (2011). Ascites. Sherlock's Diseases of the Liver and Biliary System.

[4] Markov M, Van Thiel DH, Nadir A (2007). Ascites and kidney transplantation: case report and critical appraisal of the literature. Dig Dis Sci.

[5] Hübscher SG (2011). Pathology of non-cirrhotic portal hypertension and incomplete septal cirrhosis. Diagn Histopathol.

[6] Ibarrola C, Colina F (2003). Clinicopathological features of nine cases of non-cirrhotic portal hypertension: current definitions and criteria are inadequate. Histopathology.

[7] Castro G, Freitas C, Beirão I, Rocha G, Henriques AC, Cabrita A (2008). Chylous ascites in a renal transplant recipient under sirolimus (rapamycin) treatment. Transplant Proc.

[8] Kawaguchi S, Nohara T, Shima T, Matsuyama S, Nose C, Yamahana J (2016). Massive Ascites in a Renal Transplant Patient after Laparoscopic Fenestration of a Lymphocele. Case Rep Transplant.

[9] Weber NT, Sigaroudi A, Ritter A, Boss A, Lehmann K, Goodman D (2017). Intractable ascites associated with mycophenolate in a simultaneous kidney-pancreas transplant patient: a case report. BMC Nephrol.

[10] Gane E, Portmann B, Saxena R, Wong P, Ramage J, Williams R (1994). Nodular regenerative hyperplasia of the liver graft after liver transplantation. Hepatology.

[11] Han SH, Reynolds TB, Fong TL (1998). Nephrogenic ascites. Analysis of 16 cases and review of the literature. Medicine (Baltimore).

[12] Bernard PH, Le Bail B, Cransac M, Barcina MG, Carles J, Balabaud C (1995). Progression from idiopathic portal hypertension to incomplete septal cirrhosis with liver failure requiring liver transplantation. J Hepatol.

[13] Schinoni MI, Andrade Z, de Freitas LA, Oliveira R, Paraná R (2004). Incomplete septal cirrhosis: an enigmatic disease. Liver Int.

[14] Barnett JL, Appelman HD, Moseley RH (1992). A familial form of incomplete septal cirrhosis. Gastroenterology.

[15] Schouten JN, Garcia-Pagan JC, Valla DC, Janssen HL (2011). Idiopathic noncirrhotic portal hypertension. Hepatology.

[16] Sarin SK, Kumar A, Chawla YK, Baijal SS, Dhiman RK, Jafri W, Members of the APASL Working Party on Portal Hypertension (2007). Noncirrhotic portal fibrosis/idiopathic portal hypertension: APASL recommendations for diagnosis and treatment. Hepatol Int.

[17] Dhiman RK, Chawla Y, Vasishta RK, Kakkar N, Dilawari JB, Trehan MS (2002). Non-cirrhotic portal fibrosis (idiopathic portal hypertension): experience with 151 patients and a review of the literature. J Gastroenterol Hepatol.

[18] Fan CQ, Crawford JM (2014). Sinusoidal obstruction syndrome (hepatic veno-occlusive disease). J Clin Exp Hepatol.

[19] European Association for the Study of the Liver (2016). EASL Clinical Practice Guidelines: Vascular diseases of the liver. J Hepatol.

